# Effect of water quality variation on fish assemblages in an anthropogenically impacted tropical estuary, Colombian Pacific

**DOI:** 10.1007/s11356-020-08971-2

**Published:** 2020-04-30

**Authors:** Guillermo Duque, Diego Esteban Gamboa-García, Andrés Molina, Pilar Cogua

**Affiliations:** 1 Facultad de Ingeniería y Administración, Universidad Nacional de Colombia Sede Palmira, Palmira, Valle del Cauca Colombia; 2 Facultad de Ciencias Agrarias, Universidad Nacional de Colombia Sede Palmira, Palmira, Valle del Cauca Colombia; 3 Universidad Nacional de Colombia Sede Caribe, San Andrés y Providencia, Colombia; 4 Facultad de Ciencias Básicas, Universidad Santiago de Cali, Cali, Valle del Cauca Colombia

**Keywords:** Estuarine fish, Tropical estuary, Inorganic pollution, Nitrates, Nitrites, Phosphates, Buenaventura Bay

## Abstract

In tropical estuaries, fish diversity varies spatially and temporally due to behavioral processes such as reproductive migrations, predator avoidance, and foraging, which are affected by water quality. Eutrophication is one of the main factors affecting water quality in estuaries. The objective of this study was to determine variation in fish assemblage explained by fluctuating water quality in the Buenaventura Bay. Fish were captured using artisanal trawl nets during the wet, dry, and transitional seasons at four sampling sites. Additionally, alkalinity; phosphate, nitrite, and nitrate concentrations; dissolved oxygen; pH; temperature; and suspended solids were measured. Multivariate analysis was used to assess the effect of water quality on fish assemblage. In Buenaventura Bay, the assemblage composition of *Pseudupeneus grandisquamis*, *Daector dowi*, and *Citharichthys gilberti* was affected by nitrate concentration. Moreover, large fish biomasses were associated with high nitrite concentration, intermediate salinity, and low dissolved oxygen, suggesting that these estuaries are dominated by species tolerant to poor water quality. Species richness was associated with low nitrate and phosphate concentrations, more suitable water quality indicators, and intermediate temperatures. These results suggest that the deteriorating water quality of estuaries as a result of the anthropogenic impact could increase dominance and decrease richness, resulting in structural changes of fish assemblages.

## Introduction

The geographic, biotic, and abiotic factors affect fish richness and abundance in estuaries (Brown et al. 2007). The geographic factors include connectivity, while the biotic factors include reproductive migrations, predator avoidance, and foraging (Sheaves et al. 2015) and the abiotic factors include salinity, temperature, dissolved oxygen, sediments, and nutrients, among others (Menegotto et al. 2019; Rau et al. 2019). The fluctuation of these physicochemical variables determines the water quality, influencing the dynamic of aquatic organisms and regulating the ecological processes (Ji 2008).

Water quality of estuarine ecosystems can be characterized using the concentration ranges of nitrogen, phosphorous, and dissolved oxygen, among other characteristics, which promote appropriate ecosystem functioning and support the generation of ecosystem services (Foley et al. 2015; Pouso et al. 2018). In particular, the Colombian Pacific region is strongly socioeconomically dependent on the ecosystem services for the local fish consumption and the commercialization of fishery resources (Saavedra-Díaz et al. 2016; Salas et al. 2019; Villanueva and Flores-Nava 2019). However, previous studies from this region suggest that pollutant concentrations affect benthic communities (Martínez et al. 2019) and that these pollutants are bioaccumulating in organisms of commercial interest (Duque and Cogua 2016; Gamboa-García et al. 2020) as well as in organisms at higher trophic levels (Gamboa-García et al. 2018b).

Water quality is affected by anthropogenic waste discharge, which in turn affects pollutant concentrations and physicochemical variables and, ultimately, ecological processes such as the nutrient cycles, primary production, trophic relationships, and consumer–species dynamics (Barletta et al. 2019; Jickells et al. 2017; Lemley et al. 2017; Nie et al. 2018; Warry et al. 2016). In particular, the effect of eutrophication of coastal ecosystems caused by nutrients from rivers and discharge from adjacent communities on fish assemblages remains unknown. Eutrophication may positively affect fish assemblage by increasing secondary production through a bottom-up trophic cascade or may negatively affect fish assemblage by subjecting fish to physiological stress or hypoxia (de Mutsert et al. 2016; Fong and Fong 2018; Kenworthy et al. 2016; Nelson et al. 2019; Villafañe et al. 2017; Wilkerson and Dugdale 2016).

Taking into consideration the multiple potential environmental impacts, it is critical to study estuarine biodiversity and its dynamics at different scales to understand their processes and mechanisms (Duque et al. 2018; França et al. 2011; Sheaves and Johnston 2009; Teichert et al. 2017; Vilar et al. 2013), as well as to elucidate the effects of eutrophication in these ecosystems. Species richness, abundance, and fish biomass can be measured to assess the effect of variations in nutrient concentrations in the ecosystem. Fish biomass, in particular, may be a key variable because certain species are sensitive to gaining or losing weight as a result of eutrophication (de Mutsert et al. 2016), and may affect the total of each fish population biomass as well.

We hypothesized that (i) the diversity of fishes varies among sampling seasons and sites, (ii) the abundance of the most representative fish species of the estuary can be explained by changes in water quality, and (iii) the fish species richness and fish biomass are associated with changes in water quality. The main objective of this study was to assess the effect of water quality on estuarine fish diversity, which would enable the evaluation of potential eutrophication in Buenaventura Bay.

## Materials and methods

### Study area

This study was carried out in the estuary of Buenaventura Bay at the Tropical Eastern Pacific (77° 16′ W to 3° 56′ N). The estuary spans approximately 70 km^2^ and has a 16-km-long and 5-m-deep central canal. The unique seawater inflow is known as La Bocana and is formed by Punta Bazán in the north and Punta Soldado in the south, which are approximately 1.6 km apart (Castaño 2002).

The rivers Dagua (66 m^3^s^−1^) and Anchicayá (98 m^3^s^−1^) flow into this bay (Otero 2004). Moreover, this bay has one of the highest levels of humidity and precipitation worldwide, with ~ 6980 mm of average annual rainfall and two wet seasons (from September to November and April to June) with an average monthly rainfall of 567 mm, which represents a significant freshwater source (Cantera and Blanco 2001). The access channel for ships is 9.5 and 11.3 m deep during the low and high tides, respectively; however, as a result of maintenance dredging activities and canal expansion, the depth at the channel may reach more than 16 m (Montenegro and Torres 2016).

In the estuary, there are two well-differentiated zones: the interior and the exterior bays. Within the interior bay, port activities combined with (i) waste from fishing, logging, and mining activities; (ii) discharge from rivers that flow into the bay; and (iii) domestic discharge from the same municipality have contributed to the increasing levels of potential pollutants. These pollutants are mainly wastewater which include, nitrates, nitrites, sulfates, phosphates, and coliforms, increasing the organic matter in both, during the low and high tides (IIAP 2013). In contrast, the exterior bay is influenced by a larger touristic complex and it is more marine influenced (Cantera and Blanco 2001; Palacios and Cantera 2017).

### Field sampling

In order to study the different hydroclimatic conditions of the bay, we conducted three sampling trips at different seasons. The first one during the wet season (November 2018, total month precipitation = 753.8 mm), the second one in the dry season (March 2019, total month precipitation = 321.2 mm), and the last one in the transitional season (July 2019, total month precipitation = 469.2 mm) (IDEAM 2020). The four sampling sites represent a wide range of water salinity, water quality, nutrient availability, and fish assemblage dynamics. All samples were taken at sites with water less than 8 m depth.

**Fig. 1:**
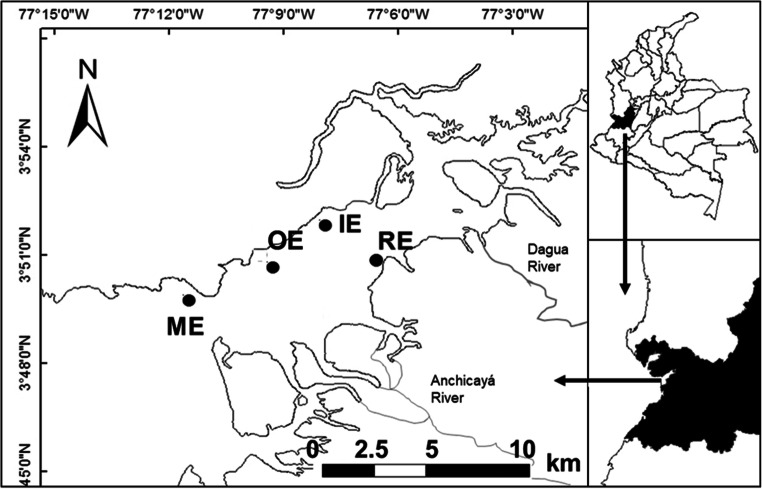
Sampling sites within the Buenaventura Bay estuary: RE = river estuary, IE = inner estuary, OE = outer estuary, ME = marine estuary

We sampled four sites within the estuary: The first one going from the inside of the bay to the outside was the river estuary (RE, 77° 6′ 33.1″ W and 3° 50′ 51.5″ N), which is the innermost site and is influenced by the Dagua River that flows into it. This site (RE) is the closest sampling site to the urban area of Buenaventura Bay, with around 300,000 inhabitants (DANE 2019). The second site was the inner estuary (IE, 77° 7′ 24.9″ W and 3° 52′ 4.4″ N), which is also located in the internal estuary but is characterized for being a little further from the river discharge and the main urban area. The third site was the outer estuary (OE, 77° 9′ 35.9″ W and 3° 50′ 58.7″ N), which is located in the external estuary and is characterized by having more compacted bottoms and further away from the main urban area. Nevertheless, this site is located near the district of La Bocana, which is inhabited by approximately 3000 people who are highly dependent on marine resources for their own consumption, for tourism (9000 a year approximately), and for supplying the main urban area markets (Escobar-Cárdenas 2009), which is the largest portion of the fish landings (no data); The fourth site was the marine estuary (ME, 77° 12′ 11.4″ W and 3° 49′ 52.44″ N), which is the outermost site and is more influenced by marine conditions and has tourism along the year (15,000 approximately). The average distance between sampling sites was 4 km (Fig. 1).

During each season and at each site, an artisanal trawling net was used for sampling with three replicates. Each trawl sampling lasted 10 min and was performed with a net with 2.54-cm mesh size and 8-m width at the mouth.

Water quality variables, including salinity, dissolved oxygen, pH, temperature, and suspended solids, were measured in situ using a multi-parameter probe YSI 556 MPS. Additionally, water samples were collected to determine alkalinity as well as nitrite, nitrate, and phosphate concentrations using a portable photometer YSI 9300.

In order to characterize the community structure, the captured fish were identified to the species level and counted, and their total length, standard length, and weight were measured. Fish identification was performed using published taxonomic keys (Fischer et al. 1995a, b; Froese and Pauly 2017; Marceniuk et al. 2017; Robertson and Allen 2015; Tavera et al. 2018).

### Data analysis

Community structure variations were assessed using species richness (i.e., number of species) and biomass (in g m^−2^) and calculated using all the captured fish. Abundance analysis was performed using only the most representative species, which were selected using the mean of the highest percent frequency (Eq. 1), abundance (Eq. 2), and weight (Eq. 3) (Martins et al. 2015).

1 $$ \mathrm{Frequency}=\left(100\times \frac{\mathrm{fish}\ \mathrm{species}\ \mathrm{presence}\ }{\mathrm{total}\ \mathrm{fish}\ \mathrm{count}}\right) $$

2 $$ \mathrm{Abundance}=\left(100\times \frac{\mathrm{number}\ \mathrm{of}\ \mathrm{fish}\ \mathrm{by}\ \mathrm{species}\ }{\mathrm{total}\ \mathrm{number}\ \mathrm{of}\ \mathrm{fish}}\right) $$

3 $$ \mathrm{Weight}=\left(100\times \frac{\mathrm{individual}\ \mathrm{fish}\ \mathrm{weight}\ \mathrm{by}\ \mathrm{species}\ }{\mathrm{total}\ \mathrm{weight}}\right) $$

The spatiotemporal analysis was addressed by calculating species richness and biomass and checking normality, using square root transformation when required. Analysis of variance was performed using season, site, and their interaction as main factors, and Tukey’s post hoc test was used to examine statistically significant differences (*p* < 0.05).

In order to assess water quality, the inorganic nitrogen was measured (nitrites and nitrates, mg L^−1^), inorganic phosphorous (phosphates mg L^−1^), and dissolved oxygen (mg L^−1^), as recommended by Lemley et al. (2015). Moreover, the analysis included salinity (PSU, practical salinity units), temperature (°C), and suspended solids (g L^−1^) measurements. Additionally, the effect of water quality on the abundances of the most important species (defined by their frequency) was calculated by a canonical correspondence analysis (CCA) using the log(*x* + 1) transformed matrix within the R environment (R Core Team 2013).

On the other hand, the variation in species richness and biomass explained by water quality was calculated by biological descriptors using Bayesian Generalized Additive Models (GAMs). The models were evaluated by using different variable combinations, and these were compared to select the best model using Akaike information criterion (AIC). For each variable, the presented models were selected considering a ∆AIC > 2 between the model and the next lowest AIC (Krause et al. 2019; Martins et al. 2015). All statistical analyses were performed within the R environment (R Core Team 2013).

It is important to mention that GAMs were used because traditional statistical methods are difficult to interpret when the variables have non-linear relationships (Rudy et al. 2016). Moreover, GAMs have been used for a wide range of applications, including medicine as well as fishery and environmental studies, among others areas of research (Amorós et al. 2018; de Souza et al. 2018; Elith et al. 2008; Tang et al. 2017).

## Results

### Spatiotemporal variation in fish assemblages

It was collected a total of 69 species belonging to 30 families. The highest species richness was observed during the transitional season at the RE (20 ± 2) and OE (20 ± 2) (*F* = 10.19, Tukey’s *p* < 0.001) sites (Table 1). However, the highest biomass (*F* = 8.69, Tukey’s *p* < 0.001) was observed during the wet season at the ME site (4.4 ± 2.4 g m^−2^) (Table 1).

**Table 1:** Fish species richness and biomass (mean ± standard deviation). Results from Tukey’s pairwise comparisons (two-way *p* ≤ 0.05) are represented with letters for each variable, which are read vertically from letters a to d. Means were calculated using three replicates. *RE*, river estuary; *IE*, inner estuary; *OE*, outer estuary; *ME*, marine estuary

Season	Site	Fish	
		Species richness	Biomass
Nov 2018 (wet)	RE	6 ± 4.6 ^a^	1.2 ± 0.1 ^abc^
	IE	13.8 ± 2.9 ^abcd^	1.8 ± 0.5 ^abcd^
	OE	9.7 ± 3.2 ^abc^	1.2 ± 0.8 ^abc^
	ME	15.7 ± 2.1 ^bcd^	4.2 ± 2.4 ^d^
March 2019 (dry)	RE	10 ± 4.0 ^abc^	1.2 ± 1.1 ^abc^
	IE	7.7 ± 1.5 ^ab^	0.7 ± 0.1 ^a^
	OE	16.7 ± 2.5 ^cd^	2.6 ± 0.8 ^abcd^
	ME	6.7 ± 4.0 ^a^	0.7 ± 0.5 ^ab^
July 2019 (transitional)	RE	20 ± 2.0 ^d^	3.8 ± 1.1 ^cd^
	IE	13.3 ± 3.8 ^abcd^	4.0 ± 0.9 ^cd^
	OE	20 ± 2.0 ^d^	3.5 ± 0.9 ^bcd^
	ME	7 ± 0.1 ^ab^	0.4 ± 0.2 ^a^

The most representative species (defined using the mean of frequency, abundance, and biomass) were *Sphoeroides trichocephalus* (57.85%), *Cathorops multiradiatus* (19.73%), *Achirus klunzingeri* (18.06%), *Lile stolifera* (16.7%), and *Pseudupeneus grandisquamis* (16.31%). The highest abundance of *Sphoeroides trichocephalus* was recorded during the transitional season at the RE site (227 ± 14.9 fish) (*F* = 2.16, Tukey’s *p* < 0.05), whereas the highest abundance of *Cathorops multiradiatus* was recorded during the wet season at the ME site (51.7 ± 35.4 fish) (*F* = 18.76, Tukey’s *p* < 0.001). In contrast, the abundance of *Achirus klunzingeri* did not show significant differences when the season and sampling site interaction was analyzed, and abundance was the highest at the OE site (*F* = 5.08, Tukey’s *p* < 0.01). Similarly, the abundance of *Lile stolifera* was the highest during the wet season at the IE site (*F* = 3.24, Tukey’s *p* < 0.05). In addition, the abundance of *Achirus klunzingeri* was the highest during the dry season at the OE site (*F* = 15.14, Tukey’s *p* < 0.001) (Table 2).

**Table 2:** Abundance of the most representative fish species of assemblages (mean ± standard deviation). Results from Tukey’s pairwise comparisons (two-way *p* ≤ 0.05) are represented with letters, which are read vertically from letters a to d, for each fish species. Means were calculated using three replicates. *RE*, river estuary; *IE*, inner estuary; *OE*, outer estuary; *ME*, marine estuary; *S_tri*, *Sphoeroides trichocephalus*; *C_mul*, *Cathorops multiradiatus*; *A_klu*, *Achirus klunzingeri*; *L_sto*, *Lile stolifera*; *P_gra*, *Pseudupeneus grandisquamis*

Season	Site	Fish species				
		S_tri	C_mul	A_klu	L_sto	P_gra
Nov 2018 (wet)	RE	139 ± 27.8 ^bcd^	1 ± 1.7 ^a^	0.7 ± 1.2	0.7 ± 0.6 ^ab^	0.3 ± 0.6 ^a^
	IE	65.7 ± 18.6 ^ab^	0.3 ± 0.6 ^a^	0.7 ± 0.6	9.3 ± 8.4 c	0.7 ± 0.6 ^ab^
	OE	51 ± 18.5 ^ab^	2 ± 1.7 a	2.3 ± 0.6		
	ME	5 ± 2.0 ^a^	51.7 ± 35.4 ^c^	0.3 ± 0.6		
March 2019 (dry)	RE	78.3 ± 135.7 ^abc^	3.7 ± 2.3 ^ab^	1.3 ± 2.3	2.7 ± 3.1 ^ab^	3.7 ± 0.6 ^b^
	IE	23.3 ± 18.5 ^ab^			0.3 ± 0.6 ^a^	2 ± 1.0 ^ab^
	OE	91.3 ± 35.2 ^abc^		0.7 ± 0.6	1 ± 1.0 ^ab^	16.3 ± 5.1 ^c^
	ME	1 ± 1.7 ^a^			0.3 ± 0.6 ^a^	2.7 ± 3.1 ^ab^
July 2019 (transitional)	RE	227 ± 14.9 ^d^	10.7 ± 3.2 ^bc^	2 ± 1.0 ^a^	8.3 ± 8.1 ^bc^	1 ± 1.0 ^ab^
	IE	121 ± 112.3 ^abcd^		1.3 ± 1.2	0.3 ± 0.6 ^a^	
	OE	193 ± 67.6 ^cd^	0.7 ± 1.2 ^a^	1.3 ± 0.6	2.3 ± 2.1 ^ab^	
	ME	23.3 ± 7.6 ^ab^	0.7 ± 0.6 ^a^			

### Spatiotemporal variation in water quality

In Buenaventura Bay, the highest salinity was recorded during the dry season (22.24 ± 2.05 PSU), followed by the transitional season (21.17 ± 1.38 PSU) and the wet season (15.83 ± 0.87 PSU) (*F* = 1212.01, Tukey’s *p* < 0.001). Moreover, spatial analysis revealed a salinity gradient, in which salinity was the lowest in the inner bay (RE and IE) and increased closer to the sea (OE and ME) (*F* = 139.51, Tukey’s *p* < 0.001). The highest salinity was recorded during the dry season at the ME site (25.56 ± 0.15 PSU) (*F* = 31.78, Tukey’s *p* < 0.001) (Table 3).

**Table 3:** Nutrient concentrations across seasons and sampling sites (mean ± standard deviation). Results from Tukey’s pairwise comparisons (two-way *p* ≤ 0.05) are represented with letters for each water quality variable, which are read vertically from letters a to d. Means were calculated using three replicates. *RE*, river estuary; *IE*, inner estuary; *OE*, outer estuary; *ME*, marine estuary. According to reports on Australian and African estuaries, nutrient eutrophication levels can be used to determine water quality. Nitrogen: optimal (< 0.1 mg L^−1^), low (0.1–1.0 mg L^−1^), and extremely low (> 1.0 mg L^−1^); inorganic phosphorous: optimal (< 0.01 mg L^−1^), low (0.01–0.1 mg L^−1^), and extremely low (> 0.1 mg L^−1^); dissolved oxygen: optimal (> 5 mg L^−1^), low (2–5 mg L^−1^), and extremely low (< 2 mg L^−1^) (Lemley et al. 2015, 2017)

		Nitrates (mg L^−1^)	Nitrites (mg L^−1^)	Phosphates (mg L^−1^)	Dissolved Oxygen (mg L^−1^)	Salinity (PSU)	Temperature (°C)
wet (Nov 2018)	RE	1.64 ± 0.17 ^cd^	0.08 ± 0.01 ^bc^	0.18 ± 0.06 ^b^	6.92 ± 0.29 ^d^	14.53 ± 0.27 ^a^	27.97 ± 0.03 ^c^
	IE	0.96 ± 0.14 ^a^	0.06 ± 0.02 ^ab^	0.15 ± 0.03 ^ab^	7.17 ± 0.39 ^d^	15.96 ± 0.50 ^b^	28.13 ± 0.08 ^cd^
	OE	1.27 ± 0.05 ^abc^	0.03 ± 0.01 ^a^	0.07 ± 0.01 ^a^	7.18 ± 0.37 ^d^	16.16 ± 0.18 ^b^	28.21 ± 0.01 ^d^
	ME	1.14 ± 0.21 ^abc^	0.06 ± 0.01 ^ab^	0.08 ± 0.02 ^a^	6.43 ± 0.81 ^bcd^	16.68 ± 0.21 ^b^	28.06 ± 0.02 ^cd^
transitional (July 2019)	RE	2.52 ± 0.01 ^ef^	0.11 ± 0.02 ^cd^	0.08 ± 0.01 ^ab^	5.48 ± 0.30 ^abc^	21.49 ± 0.87 ^de^	29.00 ± 0.11 ^f^
	IE	2.56 ± 0.03 ^f^	0.17 ± 0.01 ^e^	0.08 ^ab^	5.00 ± 0.14 ^a^	20.24 ± 0.83 cd	28.83 ± 0.03 ^f^
	OE	1.51 ± 0.13 ^bcd^	0.12 ± 0.02 ^d^	0.13 ± 0.08 ^ab^	4.93 ± 0.11 ^a^	19.95 ± 0.24 ^c^	28.96 ± 0.02 ^f^
	ME	2.02 ± 0.35 ^de^	0.10 ± 0.02 ^cd^	0.07 ± 0.02 ^a^	6 ± 0.17 ^abcd^	23.01 ± 0.39 ^f^	28.47 ± 0.09 ^e^
dry (March 2019)	RE	1.04 ± 0.03 ^ab^	0.03 ± 0.01 ^a^	0.12 ± 0.01 ^ab^	5.24 ± 0.09 ^ab^	20.54 ± 0.24 ^cde^	27.17 ± 0.07 ^b^
	**IE**	1.18 ± 0.1 ^abc^	0.04 ± 0.01 ^a^	0.15 ± 0.04 ^a^	5.35 ± 0.24 ^abc^	21.32 ± 0.39 ^de^	27.20 ± 0.09 ^b^
	OE	1.60 ± 0.17 ^cd^	0.03 ^a^	0.06 ± 0.02 ^ab^	5.51 ± 0.13 ^abc^	21.53 ± 0.13 ^e^	27.23 ± 0.10 ^b^
	ME	1.07 ± 0.1 ^ab^	0.03 ^a^	0.08 ± 0.01 ^ab^	6.50 ± 0.94 ^cd^	25.56 ± 0.15 ^g^	26.42 ± 0.02 ^a^

The mean water temperature of the Buenaventura Bay was the highest during the transitional season (28.82 ± 0.23 °C), followed by the wet season (28.09 ± 0.10 °C) and the dry season (27.01 ± 0.36 °C) (*F* = 2252.09, Tukey’s *p* < 0.001). In addition, a spatial pattern was observed, in which the mean water temperature was the lowest closer to the sea (ME) (*F* = 96.05, Tukey’s *p* < 0.001). The highest mean water temperature was recorded during the transitional season at the RE site (29.00 ± 0.11 °C), and the lowest water temperature was verified during the dry season at the ME site (26.42 ± 0.02 °C) (*F* = 27.10, Tukey’s *p* < 0.001).

Across all seasons and sampling sites, the highest concentration of dissolved oxygen was recorded during the wet season at the RE (6.92 ± 0.29 mg L^−1^), IE (7.17 ± 0.39 mg L^−1^), and OE (7.18 ± 0.37 mg L^−1^) sites (*F* = 11.41, Tukey’s *p* < 0.001). Between seasons, the highest concentration of dissolved oxygen was recorded during the wet season (6.92 ± 0.54 mg L^−1^), followed by the dry (5.65 ± 0.67 mg L^−1^) and the transitional (5.35 ± 0.48 mg L^−1^) (*F* = 115.06, Tukey’s *p* < 0.001) seasons. Spatially, the site with the highest dissolved oxygen concentration was at the ME site (6.31 ± 0.67 mg L^−1^) (*F* = 6.18, Tukey’s *p* < 0.001) (Table 3).

Considering the season and sampling site interaction, the highest concentration of nitrates was recorded during the transitional season at the IE site (2.56 ± 0.03 mg L^−1^) (*F* = 13.93, Tukey’s *p* < 0.001). Among seasons, the highest nitrate concentration was recorded during the transitional season (2.15 ± 0.47 mg L^−1^) (*F* = 92.78, Tukey’s *p* < 0.001). Spatially, the highest nitrate concentrations were recorded in the inner zone (RE = 1.73 ± 0.65 mg L^−1^, IE = 1.56 ± 0.75 mg L^−1^) and decreased toward the sites closer to the sea (OE = 1.46 ± 0.18 mg L^−1^, ME = 1.41 ± 0.5 mg L^−1^) (*F* = 5.16, Tukey’s *p* < 0.01) (Table 3).

Across all seasons and sampling sites, the highest concentration of nitrites was recorded during the transitional season at the IE site (0.17 ± 0.01 mg L^−1^) (*F* = 9.23, Tukey’s *p* < 0.001). Similarly, nitrite concentration was the highest during the transitional season (0.13 ± 0.03 mg L^−1^) (*F* = 211.63, Tukey’s *p* < 0.001) and ranged from high concentrations in the inner estuary (IE = 0.09 ± 0.06 mg L^−1^, RE = 0.074 ± 0.04 mg L^−1^) to low concentration toward the sites closer to the sea (*F* = 10.83, Tukey’s *p* < 0.001).

Along the estuary, the highest concentration of phosphates was recorded during the wet season at the RE site (0.18 ± 0.06 mg L^−1^) (*F* = 4.01, Tukey’s *p* < 0.01). Although phosphate concentration comparisons were not statistically significant across seasons (*F* = 1.94), a gradient was detected with the inner sites presenting higher concentrations (IE = 0.13 ± 0.04 mg L^−1^, RE = 0.12 ± 0.05 mg L^−1^) than the sites closer to the sea (OE = 0.09 ± 0.06 mg L^−1^, ME = 0.07 ± 0.01 mg L^−1^) (*F* = 5.36, Tukey’s *p* < 0.01) (Table 3).

### Effect of water quality variation on the abundance of the most representative fish species

The canonical correspondence analysis (CCA) suggested that the distribution of the most representative estuarine fish species and the water quality variables nitrites, nitrates, temperature, and dissolved solids were significantly correlated on the first and second ordination axes (*r* = 0.86 and *r* = 0.77, respectively), which explained 32% of the variance between fish species and water quality and physicochemical variables (Table 4). The results of the permutational test were significant (*p* = 0.001), indicating that the relationships between fish species abundance and water quality variables were significant.

**Table 4:** Canonical correspondence analysis (CCA) of the most representative estuarine fish species and water quality variables. The correlations between fish species abundance and water quality variables are indicated in italics

Total inertia			1.12
model *p* value			0.001
	CCA1	CCA2	
% of variation explained	20	12	
% of variation explained (cumulative)	20	32	
Species–environment correlations	*0.86*	*0.77*	
*p* value	0.001	0.009	
Water quality indicator variables			*p* value
Nitrites	0.52	− 0.62	0.001
Temperature	0.76	− 0.46	0.001
Total dissolved solids	− 0.37	− 0.02	0.001
Nitrates	0.22	− 0.37	0.04
pH	− 0.09	− 0.53	0.05
Phosphates	0.17	− 0.34	0.23
Alkalinity	0.40	0.07	0.24
Dissolved oxygen	0.11	0.09	0.31

**Fig. 2:**
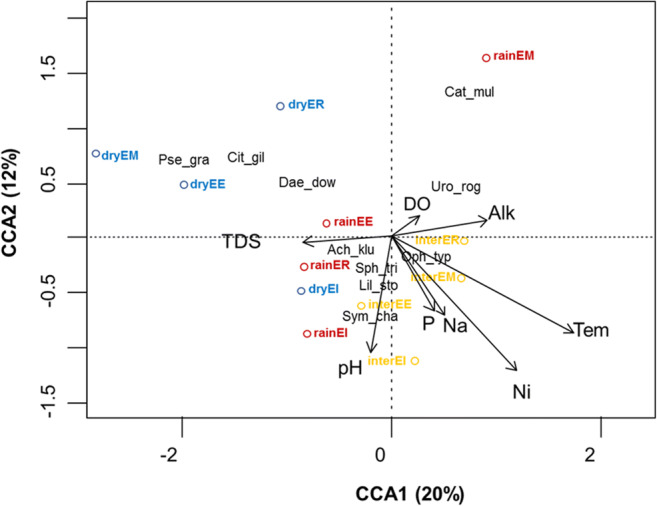
Canonical correspondence analysis ordination plot illustrating the relationships between the abundance of the most representative species with sampling sites and water quality variables. The arrows indicate water quality variables. **Alk** = alkalinity, **DO** = dissolved oxygen, **Na** = nitrates, **Ni** = nitrites, **P** = phosphates, **TDS** = total dissolved solids, **Tem** = temperature. Unfilled circles represent the combination between seasons (**dry** = dry season, **rain** = wet season, and **inter** = transitional season) and sampling sites (**RE** = river estuary, **IE** = inner estuary, **OE** = outer estuary, **ME** = marine estuary)

The first axis was positively correlated with nitrites and temperature and negatively correlated with total dissolved solids, thus representing the temporal gradient of water quality, with the dry season samples at one end and wet and transitional season samples at the other (Fig. 2 and Table 4). The second axis was negatively correlated with nitrites, temperature, and pH, thus differentiating between the seasons and sampling sites with extreme environmental conditions and the rest of the sampling sites, with the wet season at the ME site together with the dry season at the RE site at one end and the rest of the season–site combinations at the other end.

The water quality variables nitrite concentration, temperature, total dissolved solids, nitrate concentration, and pH significantly affected fish assemblage and habitat distribution comprised by the season–sampling site interactions. Moreover, the habitats comprised by the interaction of the transitional season with the OE and IE sites as well as of the wet season with the IE site displayed the highest nutrient eutrophication, pH, and temperature, although no particular fish assemblage was associated to these environmental conditions. Conversely, the environmental conditions characteristic of the interaction between the dry season and the OE, ME, and RE sites displayed the lowest nutrient eutrophication and temperatures but the highest concentration of dissolved solids, and these conditions were associated with a fish assemblage of three species (Fig. 2 and Table 4).

It was determined a fish assemblage was composed of *Sphoeroides trichocephalus*, *Lile stolifera*, *Achirus klunzingeri*, and *Ophioscion typicus*, which plotted close to the origin of the axes, suggesting that these fish species are not affected by the water quality gradient (Fig. 2). A second fish assemblage composed of *Pseudupeneus grandisquamis*, *Daector dowi*, and *Citharichthys gilberti* was associated with the dry seasons and low nitrite concentration and temperatures but high total dissolved solid concentrations (Fig. 2). Finally, a third fish assemblage composed of *Cathorops multiradiatus* and *Urotrygon rogersi* was associated with low nitrite concentration, temperatures, and pH but high dissolved oxygen concentrations (Fig. 2).

### Effects of water quality on fish species richness and biomass variation

The totality of the water quality variables was included in the univariate GAM. The total fish biomass was significantly affected by concentration of nitrates, nitrites, and total dissolved solids; salinity; temperature; and dissolved oxygen (*p* < 0.05), suggesting that each variable affects fish biomass separately but only accounts for little variation (Table 5). The largest variation was explained by concentrations of nitrates (43.4% (Adj. *R*^2^ = 0.25)) and total dissolved solids (31.4% (Adj. *R*^2^ = 0.35)), and salinity (28.9% (Adj. *R*^2^ = 0.23)), respectively. The model fit for each explanatory variable was low; therefore, a multivariate analysis was performed to assess the overall effect on fish biomass.

**Table 5:** Results of univariate generalized additive models (GAM) assessing variation in estuarine fish biomass and species richness. Model fit (Adj. *R*^2^), percentage of variation explained by each variable, as well as the polynomial grade associated with each variable. *Abbreviations*: *Alk*, alkalinity; *DO*, dissolved oxygen; *Na*, nitrates; *Ni*, nitrites; *P*, phosphates; *TDS*, total dissolved solids; *Tem*, temperature; *Sal*, salinity

Smoothing effect		S (Na)	S (Ni)	S (P)	S (Alk)	S (Sal)	S (Tem)	S (TDS)	S (pH)	S (OD)
Biomass	edf	4.72	1	1	1	2.92	1.25	3.07	3.22	1.11
	*F*-value	3.32	8.85	0.43	1.04	2.94	7.6	3.13	1.44	4.31
	*p* value	0.01*	0.005**	0.52	0.31	0.04*	0.003**	0.03*	0.28	0.03*
	Variation explained (%)	43.4	20.7	1.25	2.98	28.9	27.4	31.4	19.4	14.9
	Adj. *R*^2^	0.35	0.18	− 0.02	0.001	0.23	0.25	0.25	0.11	0.12
Species richness	edf	1	1	1	1	2.72	2.94	2.75	2.44	1.08
	*F*-value	2.34	4.14	1.9	0.1	3.62	5.01	3.74	1.2	4.89
	*p* value	0.13	0.05*	0.18	0.75	0.02*	0.003**	0.02*	0.35	0.03*
	Variation explained (%)	6.43	10.9	5.29	0.3	30.2	39.5	31	14.4	15.4
	Adj. *R*^2^	0.04	0.08	0.03	− 0.03	0.24	0.34	0.25	0.08	0.13

*p < 0.05; **p < 0.01; ***p < 0.001

On the other hand, fish species richness was significantly and individually affected by temperature; salinity; and concentrations of total solids, nitrites, and dissolved oxygen (*p* < 0.05) but explained only little variation (Table 5). The largest variation was explained by temperature (39.5% (Adj. *R*^2^ = 0.34)) and salinity (30.2% (Adj. *R*^2^ = 0.24)). Likewise, the model fit for each explanatory variable was low; therefore, a multivariate analysis was performed to assess the overall effect on fish species richness.

Multivariate analysis showed that the best model for fish biomass included nitrites and dissolved oxygen concentrations and salinity (AIC = 117.97). This model revealed a positive relationship between fish biomass and nitrate concentration and non-linear relationships between salinity (degree = 4) and dissolved oxygen concentration (degree = 2.7) and explained 64.2% of variation (Adj. *R*^2^ = 0.54). In contrast, the best multivariate model for fish species richness included nitrates, phosphates, temperature, and pH (AIC = 208.17) and revealed a negative relationship of fish species richness with phosphate and nitrate concentrations as well as a non-linear relationship with temperature (degree = 2.7) and explained 61.2% of variation (Adj. *R*^2^ = 0.52) (Table 6).

**Table 6:** Results for multivariate analysis (GAM). Results assessing variation in estuarine fish biomass and species richness. Number of species (*n*), model fit (Adj. *R*^2^ and AIC), percentage of deviance explained by each model, and the linear coefficient or polynomial grade associated with each variable. The coefficient is presented between parentheses and specifies a direction for linear relationships

	Biomass	Species richness
*N*	36	
Adj. *R*^2^	0.54	0.52
Dev. explained (%)	64.2	61.2
AIC	117.97	208.17
Coefficient or polynomial grade		
Nitrates	―	(− 4.9)*
Nitrites	1.21*	―
Phosphates	―	(− 42.1)*
Alkalinity	―	―
Salinity	4**	―
Temperature	―	2.7***
Total dissolved solids	―	―
pH	―	1.6
DO	2.7	―

*p < 0.05; **p < 0.01; ***p < 0.001; ― removed during model selection

**Fig. 3:**
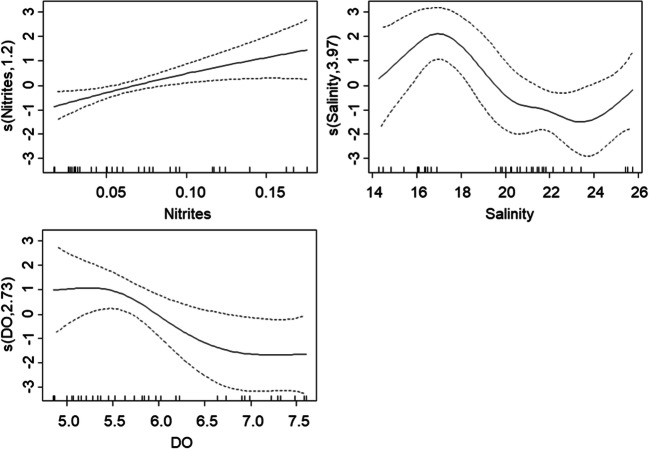
Effect of water quality predictor variation on fish biomass (multivariate analysis: GAM). Plots represent relationships indicated by the best fitting GAM **(**Table 6**)**. Smoothed functions are presented as solid lines; dashed lines denote 2 standard errors

The total fish biomass showed a positive relationship with nitrite concentration and a non-linear relationship with salinity and dissolved oxygen concentrations, peaking around 17 PSU for salinity and decreasing at 5.5 mg L^−1^ for dissolved oxygen (Fig. 3).

**Fig. 4:**
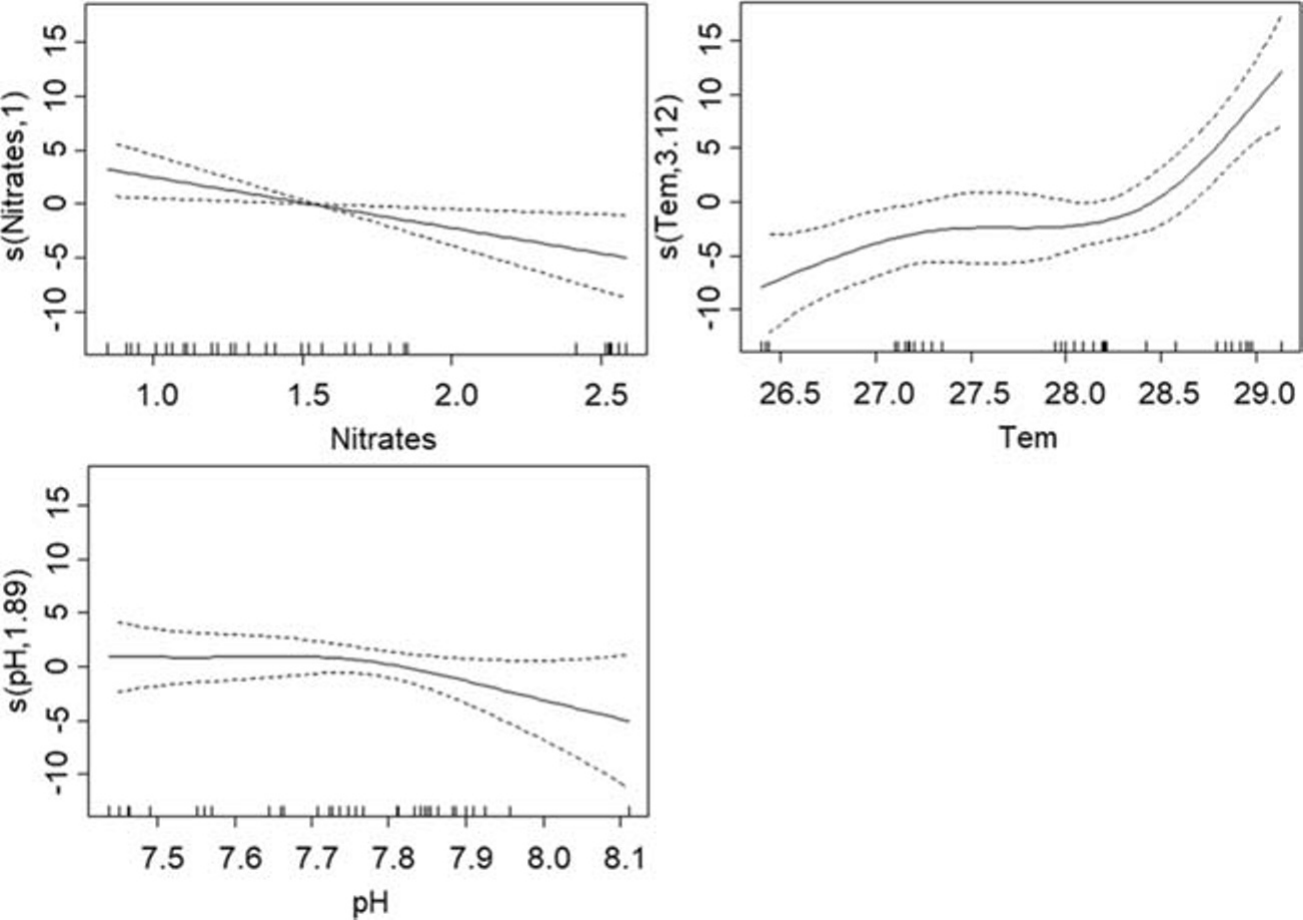
Effect of water quality predictor variation on fish species richness (multivariate analysis: GAM). Plots represent relationships indicated by the best fitting GAM (Table 6). Smoothed functions are presented as solid lines; dashed lines denote 2 standard errors

In contrast, fish species richness showed a negative relationship with nitrate concentration and was the highest at mean temperatures between 28 and 29 °C but showed no significant relationship with pH (Fig. 4).

In summary, fish biomass was the largest at higher nitrite concentrations, intermediate salinities between 16 and 18 PSU, and dissolved oxygen between 5 and 5.5 mg L^−1^, which were the lowest recorded in this study. However, fish species richness was the highest at lower nitrate and phosphate concentrations and temperatures between 28 and 29 °C.

## Discussion

### Spatiotemporal variation in fish biomass and species richness

In Buenaventura Bay, the fish assemblages varied across seasons and sampling sites. Among the 69 fish species collected, 25 (36%) accounted for 90% of the biomass, 16 (23%) were present in at least 30% of the trawling events, and 15 (22%) accounted for 90% of the abundance. The presence of dominant species has been previously reported in other studies in this region (Molina et al. 2020) as well as in other tropical estuaries (Castillo-Rivera et al. 2010) and might be explained by the tolerance of these species to a wide range of environmental conditions characteristic of these ecosystems (Sheaves et al. 2015).

In this study, the lowest species richness was recorded during the wet season in the inner estuary (*n* = 6, 8.7%) and during the dry season in the outer estuary (*n* = 7, 10.14%). The extreme salinities might explain the lower species richness, as few organisms tolerate these extremes (González-Sansón et al. 2016). This trend has been previously reported in other estuaries from the same region, where the lowest fish species richness was recorded during the seasons with the lowest salinity (*n* = 4, 6.3%) (Páez et al. 2018).

Nevertheless, the dominance of some species and the lower species richness during certain seasons and at some sampling sites could also be explained by multiple anthropogenic impacts (Fausch et al. 1990; Harrison and Whitfield 2004). In fact, other anthropogenically impacted estuaries followed similar trends. For example, in an Ecuadorian estuary characterized by high population density and mangroves disturbed by shrimp farming, four fish species (12%) accounted for 90% of the fish abundance (Shervette et al. 2007). Similarly, in a Mexican coastal lagoon characterized by high population density and tourist activities, only eight fish species (12.5%) accounted for 90% of the fish abundance (Páez et al. 2018). In the Buenaventura Bay, 25 fish species (36%) accounted for 90% of the fish abundance, suggesting that this ecosystem is resilient to the multiple anthropogenic disturbances. Nonetheless, dominant species might thrive in highly disturbed estuarine ecosystems, thus threatening biodiversity within these ecosystems.

On the other hand, the most abundant fish species was *Sphoeroides trichocephalus* (Tetraodontidae), in particular during the transitional season and at sites with contrasting characteristics: river discharge (RE) and compacted bottoms (OE). The transitional season corresponds to the July month, which is one of the periods of highest flux of tourism in the Dagua basin and Bocana sand beaches (Herrera et al. 2007; Ospina Niño 2017). The Dagua River drains into the RE site, and Bocana is close to the OE site, which may suggest that during this time there is an increase in anthropogenic discharges over these areas, which, paradoxically, is related to higher abundances of *S. trichocephalus*. Moreover, the wide environmental distribution of this species may be explained by the differential niche use of juvenile and adult fish (Velasco and Wolff 2000). Juveniles might benefit from murkier waters for predator avoidance, while adults might exploit multiple bottom types for foraging. Finally, *S. trichocephalus* has been reported to tolerate extreme environmental conditions, which allows it to occupy most of the available habitats within the estuary throughout the year (Molina et al. 2020).

### Spatiotemporal variation in water quality

In the studied site, it was recorded a temporal gradient in which salinity was the highest during the dry season, followed by the transitional and wet seasons, as well as a spatial gradient in which salinity was the lowest in the inner sites and highest in the outer sites. This pattern has been previously reported in studies in the same bay and was characterized by salinities below 26 PSU due to high rainfall and runoff from the Dagua and Anchicayá Rivers (Cantera et al. 1999; Gamboa-García et al. 2018a, 2020; Molina et al. 2020).

Nitrate and nitrite concentrations were the highest during the transitional season and at the inner sites, whereas phosphate concentration was the highest during the wet season and at the inner sites. During seasons with the highest rainfall, erosion and runoff increase the discharge of nutrients of the organic matter from mangroves, as well as the anthropogenic runoff that flows into river basins (Nie et al. 2018), in this case the Dagua and Anchicayá Rivers, which might explain the observed patterns. The Dagua River basin, which includes the municipalities of Dagua and Buenaventura, is characterized by anthropogenic pressures including human settlements, tourism activities, farming, and the consequent use of fertilizers and mining, among others. Moreover, the inner areas of the estuary are directly affected by domestic wastewater runoff from the Buenaventura Bay. Additionally, pollution and mangrove logging impacts have been reported upstream of the mouth of the Dagua River (Cantera et al. 1999; Romero et al. 2006), which could have an effect on nutrient cycling in these ecosystems. This agrees with reports from northern Brazil (Goiana River estuary), where the highest phosphorus concentration was reported during the season with the highest rainfall and within the inner estuary (Costa et al. 2017).

In this study, most of the sites and seasons presented a moderate to low water quality. For example, the lowest dissolved oxygen concentrations were recorded during the transitional season and at the IE (5.00 ± 0.14 mg L^−1^) and OE (4.93 ± 0.11) sites, which were classified as moderate. However, these sites were classified as having low water quality due to their nutrient and inorganic phosphorus concentrations. These results highlight the susceptibility to low water quality along the Buenaventura Bay estuary and during the year. Nevertheless, a study in the Tumaco Bay (Colombian Pacific, closer to Ecuador) reported that phosphate concentration had a range of 0.2 ± 0.1 mg L^−1^ and nitrite plus nitrate concentrations of 1.9 ± 1.8 mg L^−1^ (Guzmán et al. 2014), which were similar to the ranges found in Buenaventura Bay. In that study, a phytoplankton characterization was performed, and an oceanographic analysis, which suggested that despite the susceptibility of the Tumaco Bay, water quality was improved by the hydrodynamics of the system, which may flow away the pollutants. Therefore, the variation in the water quality and the resilience of the fish community in Buenaventura Bay may be explained by its hydrodynamic regime, the prominent tide range, and the high seasonal variation of river flow, which, similar to Tumaco bay, may improve the ecosystem services.

### Effect of water quality on variations in assemblage of the most representative fish species

In the estuary of Buenaventura Bay, the most representative fish species were distributed across three assemblages according to water quality variables. The dry season on one side, and wet and transitional seasons on the other, strongly affected fish assemblage structure. The environmental variables that were correlated most strongly with the fish assemblages were mean nitrite concentration and temperature. One assemblage was composed of *Sphoeroides trichocephalus*, *Lile stolifera*, *Achirus klunzingeri*, and *Ophioscion typicus*. These species were recorded across seasons and sampling sites in more than 45% of the trawling events, suggesting that these fish species are tolerant of water quality variation. This trend has been previously reported for the estuarine resident species in Buenaventura Bay (Molina et al. 2020), as well as in other estuaries around the world (Cabral et al. 2011; Franco et al. 2006; Martinho et al. 2007). In general, species with a wide physiological tolerance breadth tend to predominate in these ecosystems (Potter et al. 2015). For example, *Sphoeroides annulatus* is classified as euryhaline and tends to dominate a great proportion of assemblages it is part of (Chávez Sánchez et al. 2008). Interestingly, this trend has also observed in the Buenaventura Bay as this species represented the highest abundance during the wet and transitional seasons and was tolerant to nutrient concentration variation, dissolved oxygen, and temperature changes.

On the other hand, a second fish assemblage composed of *Pseudupeneus grandisquamis*, *Daector dowi*, and *Citharichthys gilberti* was the most susceptible to nitrite concentration and temperature, and was only reported during the dry season. Previous studies have reported the effect of inorganic nitrogen concentration (Wilkerson and Dugdale 2016) as well as temperature on fish assemblages (Harrison and Whitfield 2006; Molina et al. 2020; Rau et al. 2019). Even though these species were only recorded during the season with the highest salinity, they were also classified as being highly dependent on bottom characteristics considering their movement and foraging behavior (Ramírez-Luna et al. 2008; Rau et al. 2019). Therefore, the increasing nitrite concentration in combination with increasing temperatures due to solar radiation could result in a bottom-up nutrient control, which could increase the eutrophication conditions and negatively affect the fish assemblages. Moreover, increased nitrite concentration (Camargo and Alonso 2006; Schlacher et al. 2007) and water temperature (Jeffries et al. 2016; Madeira et al. 2016) could represent physiologically stressful surroundings for fishes.

Furthermore, a third fish assemblage of fishes composed of *Cathorops multiradiatus* and *Urotrygon rogersi* was recorded during the wet season in the outer bay and was associated with low pH and high dissolved oxygen concentrations, which is characteristic of the runoff of the rivers Dagua and Anchicayá (Cantera and Blanco 2001). The distribution of species forming this assemblage is consistent with that reported in previous studies from this region (Castellanos-Galindo et al. 2006; Molina et al. 2020). Moreover, the highest abundance of *Cathorops multiradiatus* and *Urotrygon rogersi* recorded in the outer estuary region might be explained by the runoff of the rivers that creates environmental conditions to adjacent waters, facilitating resource provisioning to the most marine species (Elliott et al. 2007; Potter et al. 2015; Molina et al. 2020). Finally, fish assemblages varied mostly temporally as an effect of nitrite concentration and temperature, suggesting that water quality and estuarine ecosystem services in the Buenaventura Bay are susceptible to eutrophication and highlighting the complexity and ecological relevance of the processes.

### Effect of water quality on fish species richness and biomass variation

In the estuary of Buenaventura Bay, fish species richness and biomass were affected by water quality. Higher biomasses were recorded in low-quality waters, enriched with nitrites and with a low dissolved oxygen. This trend was reported during a hypoxia event in a Mexican estuary, where the fish species representing the highest biomass benefited from the bottom-up effect as a result of primary and secondary production and could also tolerate low dissolved oxygen concentrations, which allowed them to avoid predators (de Mutsert et al. 2016). In summary, in the Buenaventura Bay, the increased fish biomass and dominance of certain species could be an indicator of the effect of low water quality.

In contrast, fish species richness was the highest at intermediate salinities, which is consistent with results from the Málaga Bay, an adjacent estuary to the Buenaventura Bay, where the highest species richness was recorded at intermediate salinities (Castellanos-Galindo and Krumme 2015). These open estuaries are characterized by a wide salinity range: low-salinity habitats (under 10 PSU) that are unsuitable for marine fish in some reported estuaries (Martino and Able 2003), as well as intermediate-salinity habitats. Thus, these relatively intermediate salinities could provide a salinity ecotone, which could be tolerated by resident estuarine species as well as marine species that also depend on the estuary ontogenetically.

Furthermore, a low species richness was reported in low-quality waters, characterized by high nitrate and phosphate concentrations. Increased nitrate and phosphate concentrations have been previously linked to anthropogenic activities (Camargo and Alonso 2006; Smith 2003; Wilkerson and Dugdale 2016), such as wastewater runoff from urban settlements into estuaries, which in turn affects ecological cycles (Berbel et al. 2015). This suggests that the presence of these nutrients indicates a disturbed and low-quality habitat as a result of urban wastewater runoff, which contains nutrients as well as other pollutants. Consequently, fish could suffer from pathologies of different organs, such as the gills, liver, and kidney, or become more susceptible to parasites (Schlacher et al. 2007). Moreover, this could affect their vitality, affecting community structure and ecosystem functioning. Thus, the effect of anthropogenic nutrient runoff on fish assemblages may be evident in Buenaventura Bay, particularly in relation to nitrates, as this nutrient presented the highest concentrations in most of the estuary.

Considering the socioeconomic importance of the Buenaventura Bay estuary for the region for tourism and fishing for livelihood and commercial purposes, it is critical to monitor, control, and treat anthropogenic runoff that might flow into the estuary. In addition, the relevant authorities should develop initiatives to monitor and assess septic tanks from rural communities and tourist centers adjacent to the sea. This study highlights the importance of assessing inorganic pollution within estuaries, and future studies should complement this with histopathology of fish and its potential effect on human health, in addition, chlorophyll-a and microbiological analyses. Moreover, fish assemblages could be used as ecosystem functioning indicators, and certain fish populations should be permanent monitored.

## Data Availability

The data in this study are available upon reasonable request to the corresponding author.
